# CQ sensitizes human pancreatic cancer cells to gemcitabine through the lysosomal apoptotic pathway via reactive oxygen species

**DOI:** 10.1002/1878-0261.12179

**Published:** 2018-03-13

**Authors:** Zhiping Fu, Xi Cheng, Jie Kuang, Haoran Feng, Lingxie Chen, Juyong Liang, Xiaonan Shen, Stanley Yuen, Chenghong Peng, Baiyong Shen, Zhijian Jin, Weihua Qiu

**Affiliations:** ^1^ Department of General Surgery Ruijin Hospital Shanghai Jiao Tong University School of Medicine Shanghai China; ^2^ Shanghai Institute of Digestive Surgery Ruijin Hospital Shanghai Jiao Tong University School of Medicine Shanghai China; ^3^ Biology Chemistry Major University At Albany New York NY USA

**Keywords:** apoptosis, chloroquine, gemcitabine, lysosomal membrane permeabilization, reactive oxygen species

## Abstract

As an established anticancer drug, gemcitabine (GEM) is an effective systemic treatment for advanced pancreatic cancer (PC). However, little is known about the potential effectors that may modify tumour cell sensitivity towards GEM. Autophagy, as a physiological cellular mechanism, is involved in both cell survival and cell death. In this study, we found that exposure to GEM induced a significant increase in autophagy in a dose‐dependent manner in PANC‐1 and BxPC‐3 cells. Inhibition of autophagy by chloroquine (CQ) and ATG7 siRNA increased GEM‐induced cytotoxicity, and CQ was more effective than ATG7 siRNA. Moreover, CQ significantly enhanced GEM‐induced apoptosis, while ATG7 siRNA failed to show the similar effect. Subsequently, we identified a potential mechanism of this cooperative interaction by showing that GEM with CQ pretreatment markedly triggered reactive oxygen species (ROS) boost and then increased lysosomal membrane permeability. Consequently, cathepsins released from lysosome into the cytoplasm induced apoptosis. We showed that CQ could enhance PC cells response to GEM in xenograft models. In conclusion, our data showed that CQ sensitized PC cells to GEM through the lysosomal apoptotic pathway via ROS. Thus, CQ as a potential adjuvant to GEM might represent an attractive therapeutic strategy for PC treatment.

**Abbreviation**
AO: acridine orange
CA‐074 Me: CA‐074 methyl ester
CQ: chloroquine
Ct: cycle threshold
CTSB: cathepsin B
CTSD: cathepsin D
GEM: gemcitabine
IF: immunofluorescent
LMP: lysosomal membrane permeabilization
NAC: N‐acetyl‐l‐cysteine
PC: pancreatic cancer
pep‐A: pepstatin A
PI: propidium iodide
ROS: reactive oxygen species
RT‐PCR: quantitative real‐time PCR
TEM: transmission electron microscopy

## 1 Introduction

Pancreatic cancer (PC) is one of the most lethal human malignancies, ranking fourth in the number of cancer‐related deaths worldwide (McGuire, 2016), and the overall incidence and mortality rate of PC have shown a growth trend (Chen *et al*., 2016). PC is highly lethal with an average overall 5‐year survival rate of <5% (Sarkar *et al*., 2007), due to the lack of effective therapeutic drugs and the difficulty for early detection along with complete surgical resection. Gemcitabine (GEM) has been considered a standard chemotherapeutic first‐line treatment option of patients with PC (Hidalgo, 2010). However, resistance to GEM has been increasing in recent years, and potential effectors that may modify tumour cell sensitivity towards GEM remain unknown (Oettle *et al*., 2007). Numerous clinical trials have demonstrated few improvements in overall survival with GEM combined with other agents, including cetuximab and erlotinib (Antoniou *et al*., 2014).

Autophagy is an evolutionary conserved catabolic process that degrades cellular organelles and proteins and sustains cellular metabolism during starvation or metabolic stress (Mathew *et al*., 2007). The role of autophagy in cancer is complex, and genetic evidence has shown that basal autophagy may have a suppressive role during tumour initiation (Ding *et al*., 2008). Recent studies suggest that autophagy may play a positive role in cancer cell proliferation and may cope with various environmental stress (Degenhardt *et al*., 2006; Kuma *et al*., 2004). Autophagy may be crucial not only in the regulation of cancer development and progression, but also in determining the response of tumour cells to anticancer therapy (Apel *et al*., 2009; Yousefi and Simon, 2009). Chloroquine (CQ), an antimalarial lysosomal inhibitor, has been identified as an inhibitor of autophagy (Chen *et al*., 2011). CQ can prevent autophagy by blocking autophagosomal–lysosomal fusion events (Hasanain *et al*., 2015). Combined with many other agents, CQ has been proven to elevate the efficiency of cancer therapy in several studies (Kimura *et al*., 2013). However, the mechanism of CQ‐induced tumour cell death is poorly defined. More interestingly, recent studies have shown that CQ is capable of enhancing the cytotoxicity of antitumour drugs, independent of its inhibition of autophagy (Al‐Bari, 2015).

In this study, we found that GEM significantly induced autophagy, and the inhibition of autophagy enhanced its cytotoxicity. We also found that CQ pretreatment could enhance GEM‐induced apoptosis; however, ATG7 siRNA did not have the similar effect. In‐depth studies have demonstrated that GEM combined with CQ increased ROS and damaged lysosomal membrane, increasing lysosomal membrane permeability, followed by cathepsins releasing from lysosome into the cytoplasm, and finally inducing apoptosis. These observations indicated that CQ could be a potential adjuvant with GEM in the treatment of PC.

## 2 Materials and methods

### 2.1 Cell culture and drug treatment

Human PC cell lines PANC‐1 and BxPC‐3 were obtained from ATCC and cultured in DMEM supplemented with 10% fetal bovine serum (Gibco, New York, NY, USA) at 37 °C in a 5% CO_2_. GEM (G6423, Sigma‐Aldrich, St. Louis, MO, USA) was dissolved in DMSO and diluted with DMEM to achieve a desired concentration. Prior to each treatment, cells were plated overnight and were at similar subconfluent density at the time of drug exposure.

### 2.2 Cell viability assay

Cell viability was estimated using CCK‐8 colorimetric assay (Dojindo, Kumamoto, Japan). PANC‐1 and BxPC‐3 cells were plated in 96‐well plates at 3000 cells per well in a volume of 100 μL 24 h before treatment. The logarithmically growing cells were treated with GEM at indicated concentrations. DMSO was added to cultures as solvent control. Cells in six replicated wells were fixed with CCK8 and incubated at 37 °C for 3 h. Absorbance was read spectrophotometrically at 450 nm with a reference at 650 nm using a microtitre plate reader (Tecan Safire 2, Tecan, Switzerland). Cell viability was calculated according to the following formula: Cell proliferation (%) = [(A450sample‐A450blank)/(A450control‐A450blank)−1]*100%.

### 2.3 Immunofluorescent assay

After treatment, PANC‐1 cells were fixed with 4% paraformaldehyde for 20 min and made permeable with 0.1% Triton X‐100 at room temperature for 30 min. Cells were blocked using 5% BSA and were then incubated with primary rabbit polyclonal anti‐LC3 (1 : 100, Cell Signaling Technology, Danvers, MA, USA) and mouse mononclonal anti‐p62/SQSTM‐1 (1 : 100, Abcam, Cambridge, MA, USA) antibodies. Goat anti‐rabbit IgG‐FITC and anti‐mouse IgG‐FITC secondary antibodies were utilized for fluorescent coloration. After counterstained with DAPI, immunofluorescent images were collected using a fluorescence microscope (Olymplus, IX71, Olympus, Tokyo, Japan).

### 2.4 Quantitative real‐time PCR

Steady‐state LC3 mRNA levels were determined by real‐time PCR (RT‐PCR) using SYBR Premix Ex Taq (Takara, Tokyo, Japan). Isolation of total RNA and reverse transcription of cDNA were performed as described previously (Qiu *et al*., 2007). GAPDH was used as an endogenous control for the standardization of differences in the amounts of total RNA in each sample. The primer pairs utilized were as follows: LC3, 5′‐TACGAGCAGGAGAAAGACGAGG‐3′ and 5′‐GGCAGAG TAGGTGGGTTGGTG ‐3′; and GAPDH, 5′‐GAAGGTGAAGGTCGGAGTC‐3′ and 5′‐GAAGATGGTGATGGGATTTC‐3′. Thermal cycling was performed with 7000 Sequence Detection System (Applied Biosystems, Carlsbad, CA, USA). Reactions were carried out in a 20 μL reaction volume. PCR amplification conditions were as follows: 95 °C for 10 min, 40 cycles of 95 °C for 5 s and 60 °C for 30 s. The cycle threshold (*C*_t_) data were obtained automatically, and each data point was performed in triplicate.

### 2.5 Autophagy inhibition by RNA interference

ATG7 is essential for autophagy conjugation system and autophagosome formation (Gonzalez *et al*., 2014). To inhibit autophagy, ATG7 siRNA was transfected into PANC‐1 and BxPC‐3 cells. The ATG7‐targeting sense sequence and the universal negative control siRNA were purchased from Invitrogen (12935‐400, Invitrogen, Carlsbad, CA, USA). The human ATG7 sequence (5′‐GGAGTCACAGCTCTTCCTT‐3′) was cloned into the *Bam*H1 and *Eco*R1 sites of pGSU6‐GFP vector (GTP600300, Genlantis, San Diego, CA, USA). siRNA plasmids were transfected using Lipofectamine 2000 (11668019, Invitrogen). Forty‐eight hours after transfection, cells were collected by flow cytometry sorting. The irrelevant nucleotides that did not target any annotated human genes were served as negative control. Then, the cells were cultured and treated by GEM (20 μm) for an additional 48 h.

### 2.6 Electron microscopy analyses

After treatment, PANC‐1 cells were immediately fixed in 2% glutaraldehyde and 2.0% paraformaldehyde in 0.1 mol·L^−1^ sodium phosphate buffer (pH 7.4) at 4 °C for 3 h. After postfixed in 1% osmium tetroxide in the same buffer for 2 h and gradual dehydration in alcohols, the samples were embedded in a mixture of Epon 618 and epoxypropane. Semithin sections were stained with toluidine blue, and ultrathin sections were stained with 5% uranyl acetate and Reynold's lead citrate. Sections were examined on a Hitachi electron microscope equipped with digital camera. For morphometric analysis, at least three independent experiments were performed.

### 2.7 Apoptosis detection and cell cycle analyses

The PANC‐1 and BxPC‐3 cells’ apoptotic status after treatment was evaluated by measuring phosphatidylserine exposure on cell membranes using Annexin V‐fluorescein isothiocyanate (Annexin V‐FITC) and propidium iodide (PI) staining via BD Pharmingen Annexin V‐FITC Apoptosis Detection Kit I (BD Biosciences, San Jose, CA, USA). At indicated intervals, both attached cells and floating cells were harvested and washed twice with cold PBS and suspended in 100 μL PBS. Cells were incubated with 3 μL Annexin V‐FITC and 5 μL PI at room temperature for 15 min in the dark. An additional 300 μL of 1× binding buffer was added to each tube. Samples were analysed immediately by flow cytometry (FACScalibur, Becton Dickinson, Franklin Lakes, NJ, USA). A total of 10 000 events were acquired using green channel FL1 for Annexin V‐FITC and red channel FL3 for PI. (AnV^+^) PI^‐^ cells were considered as early apoptotic, and (AnV^+^) PI^+^ cells were considered as late apoptotic and necrotic. Both subpopulations were counted together and expressed as the total fraction of apoptotic cells. Data were analysed using cell quest software (Becton Dickinson).

Cell cycle distribution was further analysed by flow cytometry. After treatment, cells were harvested and suspended in ice‐cold PBS. Single‐cell suspensions were fixed with 75% ice‐cold ethanol at 4 °C overnight. Samples were then washed twice with PBS and incubated with PI (50 μg·mL^−1^) for 30 min in the dark and analysed by flow cytometer. The percentage of cells at each phase of the cell cycle was determined using the cell quest software (Becton Dickinson).

### 2.8 Isolation of proteins and immunoblot analyses

After treatment, cells were harvested at various time intervals and digested in RIPA buffer with presence of Protease Inhibitor Cocktail (Pierce, Rockford, IL, USA), and protein concentration was quantified using the BCA Protein Assay Kit (Pierce). After separated by SDS/PAGE, proteins were transferred to PVDF membrane (Bio‐Rad, Hercules, CA, USA). Blots were probed with anti‐LC3, anti‐p62/SQSTM‐1, anti‐CTSB, anti‐CTSD, anti‐Bcl‐2, anti‐Bax, anti‐cleaved caspase‐3 (all from Cell Signaling Technology), anti‐LAMP‐1, anti‐β‐tubulin, and anti‐GAPDH (Abcam). The goat anti‐rabbit or goat anti‐mouse horseradish peroxidase‐conjugated IgG was used as secondary antibodies (Santa Cruz Biotechnology, Dallas, TX, USA). Bands were observed by enhanced chemiluminescence (Pierce). Band density was measured by densitometry, quantified using the public domain nih image software (open source imagej software available at http://rsb.info.nih.gov/ij/) and normalized to an indicated sample in the identical membrane.

### 2.9 Determination of intracellular ROS levels

The generation of ROS in cells was evaluated by a fluorometry assay using intracellular oxidation of nonfluorescent probe 2, 7‐dichlorofluorescein diacetate (DCFH‐DA). DCFH‐DA can passively diffuse into cells and be deacetylated by esterase to form nonfluorescent 2,7‐dichlorofluorescein (DCFH). In the presence of ROS, DCFH reacts with ROS to form the fluorescent product DCF, which is trapped inside the cells. When the membrane is oxidized and damaged, the fluorescence will attenuate significantly. PANC‐1 and BxPC‐3 cells were incubated with corresponding treatment in a 6‐well plate with 3 × 10^5^ cells/well; 30 min before the end of treatment, the cells were incubated with DCFH‐DA at 37 °C for 20 min. After treatment as mentioned above, DCF fluorescence intensity was detected by fluorescence spectrometry (Spectramax Gemini, Molecular Devices, San Jose, CA, USA) with an excitation wavelength of 490 nm and an emission wavelength of 530 nm. The results were expressed as relative fluorescence intensity per 10^4^ cells.

### 2.10 Measurement of LMP

LMP was assessed by acridine orange (AO) staining. Cells were incubated in 1 mmol/L of lysosomotropic metachromatic fluorophore AO for 20 min at 37 °C. And then cells were washed and resuspended in PBS. Changes in LMP were detected by flow cytometer.

### 2.11 Cytosolic cathepsin activity assay

Assay of cathepsin B (CTSB) and cathepsin D (CTSD) activities was carried out using CTSB assay kit (abcam, ab65300) and CTSD assay kit (abcam, ab65302) according to the manufacture's protocol. Briefly, 50 μg of cytosolic fraction protein was added up to the final reactant to 200 μL per well in a 96‐well plate. Next, samples were treated with 140 mm site‐specific substrates in assay buffer at 37 °C with 10 mm DTT for 0.5 h and then analysed by flow cytometer.

### 2.12 *In vivo* PC xenograft tumour model

Four‐week‐old male BALB/c nude mice were purchased from the Institute of Zoology, Chinese Academy of Sciences of Shanghai. All experiments were performed in accordance with the official recommendations of the Chinese Zoological Society, and animals received human care according to the criteria outlined in the ‘Guide for the Care and Use of Laboratory Animals’. The suspension, containing 1 × 10^6^ PANC‐1 cells, was subcutaneously injected into the right flank of nude mice. After 2 weeks, when tumour reached around 5 mm, the mice were randomly divided into four groups (four in each group). Group 1: Vector (0.9% physiological saline), group 2: CQ (60 mg·kg^−1^), group 3: GEM (20 mg·kg^−1^) and group 4: CQ (60 mg·kg^−1^) + GEM (20 mg·kg^−1^). These compounds, dissolving in 0.9% physiological saline, were administered intraperitoneally twice per week. Tumour size and body mass were recorded twice per week. Three days after the last injection, the animals were euthanized by cervical decapitation and tumours were removed and weighed. Tumour dimensions were measured using a digital calliper, and the tumour volume was calculated using the following formula: *V* = π/6 × (*W* ^2^ × *L*).

### 2.13 Immunohistochemistry and TUNEL Assay

The immunohistochemistry of xenograft tumour in nude mice was conducted as previously described (Ma *et al*., 2016). Briefly, paraffin‐embedded tumour specimens underwent a heat pretreatment of 60 °C for 1 h, then dewaxed in xylene, rehydrated in a series of ethanol and treated with 0.01 mol·L^−1^ citrate buffer (pH 6.0) for antigen retrieval. After inhibition of endogenous peroxidase activity for 30 min with methanol containing 0.3% H_2_O_2_, the sections were stained with anti‐caspase‐3 (1 : 200, Santa Cruz) and anti‐Ki67(1 : 200, Santa Cruz) antibody at 4 °C overnight,followed with incubation by HRP‐labelled secondary antibody. The apoptotic cells were detected by TUNEL assay using In Situ Cell Death Detection Kit, Fluorescein (Roche Applied Science, Indianapolis, IN, USA), according to the manufacturer's protocol, and nuclei were detected by DAPI staining.

### 2.14 Statistical analyses

Data were expressed as means ± SD. An analysis of variance (ANOVA) and Student's *t‐*test were used for comparison among groups. The Mann–Whitney *U*‐test was used for comparison of tumour volume. Categorical data were evaluated with the *chi*‐square test or Fisher's exact test. A *P*‐value less than 0.05 was considered to be significant. Statistical analyses were analysed using spss13.0 (SPSS Inc. Chicago, IL, USA).

## 3 Results

### 3.1 GEM induces cell autophagy in PANC‐1 and BxPC‐3 cells

**Figure 1:**
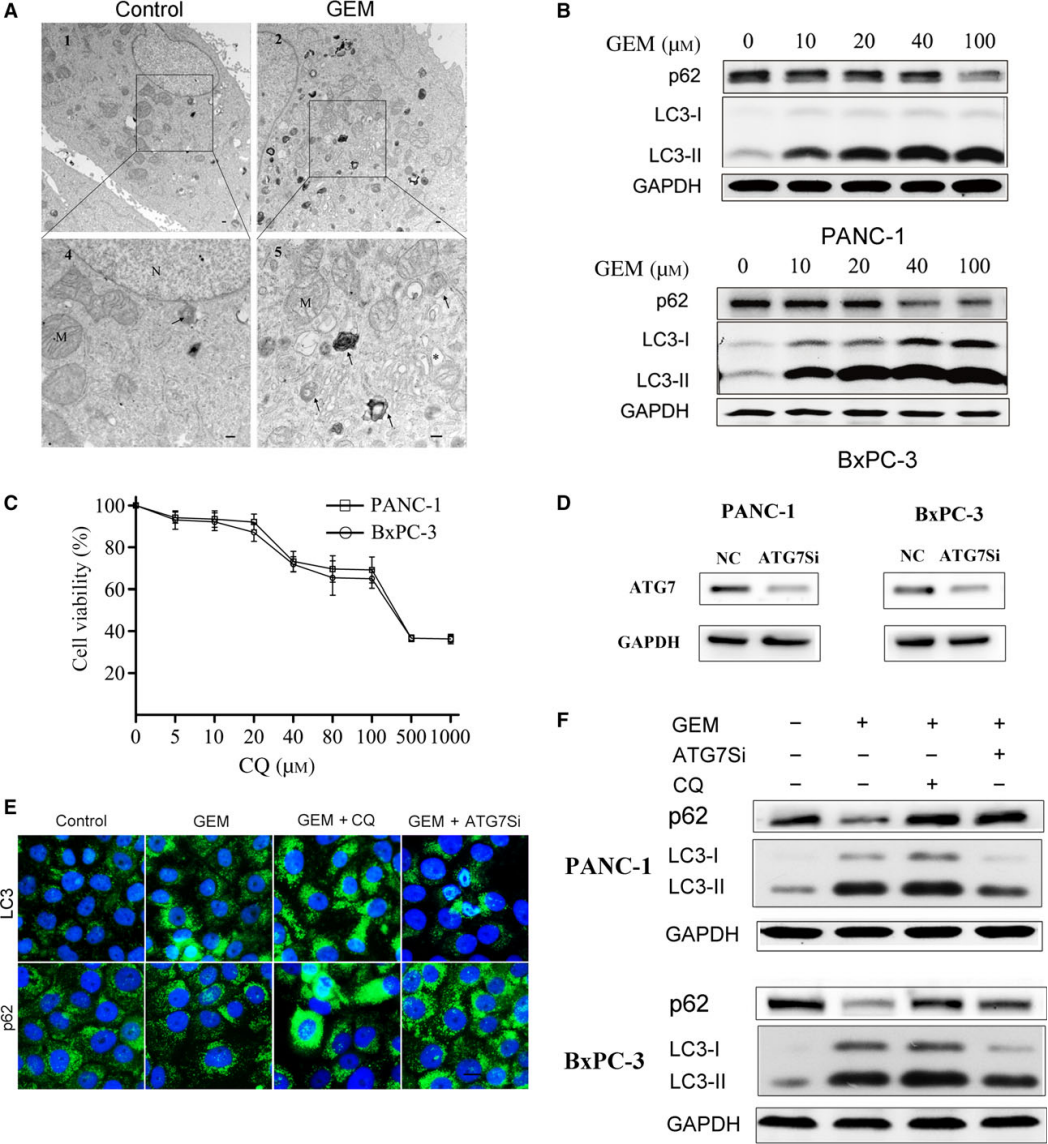
Gemcitabine‐induced autophagy could be inhibited by CQ and ATG7 siRNA. (A) Transmission electron microscopy images of PANC‐1 cells treated by GEM (20 μm) for 48 h. Scale bars are 2 μm. Arrows: dense AVs and lysosomes; N: nucleolus; M: mitochondria; asterisks: lipid vacuole. (B) PANC‐1 and BxPC‐3 cells were treated with a series of concentrations of GEM. Levels of p62 and LC3 were analysed by western blot. (C) PANC‐1 and BxPC‐3 cells were treated with different concentrations of CQ (0, 5, 10, 20, 40, 80, 100 and 1000 μm) for 24 h. (D) PANC‐1 and BxPC‐3 cells were transfected with 4 μg of NC siRNA and ATG7 siRNA, respectively, and then were treated with 20 μm of GEM for 48 h. The knock‐down effects of ATG7 were confirmed by western blot. (E) PANC‐1 cells were treated with CQ (10 μm) or ATG7 siRNA and then with GEM (20 μm), and the formation of autophagic vacuoles was determined by immunofluorescent staining for LC3 and p62. (F) PANC‐1 and BxPC‐3 cells were pretreated with ATG7 siRNA or CQ (10 μm) and then were treated with GEM (20 μm) for additional 48 h, and immunoblot analysis of LC3 and p62 was presented.

Autophagy is involved in the process of cell survival and cell death and can be induced by many cytotoxic compounds. To determine whether GEM could induce autophagy, we utilized transmission electron microscopy (TEM) to analyse the ultrastructure of PC cells treated by GEM. Autophagosomes were clearly observed in PANC‐1 cells after treatment with 20 μmol·L^−1^ GEM for 48 h, and the number of autophagosomes was greater than that of the untreated cells (Fig. 1A). LC3‐I conversion to LC3‐II is an important step in autophagy, and the number of LC3‐II puncta represents the number of autophagosomes. Western blot analysis showed that GEM significantly enhanced the expression of LC3‐II, even at the concentration of 10 μmol·L^−1^ (Fig. 1B). Moreover, the reduction in p62/SQSTM‐1, a scaffold protein known to be degraded by autophagy (Bjorkoy *et al*., 2009), was found in the two GEM‐treated PC cells (Fig. 1B).

To assess the role of GEM‐induced autophagy in PC cells, we utilized CQ, a late autophagy inhibitor. CQ concentrations ranging from 5 to 20 μmol·L^−1^ did not significantly affect cell viability, while CQ‐induced cytotoxicity increased dramatically at dosages greater than 40 μmol·L^−1^ (Fig. 1C). Therefore, the working concentration of CQ was defined at 10 μmol·L^−1^, which did not result in obvious cell necrosis. Moreover, ATG7 siRNA was utilized as another method to inhibit autophagy. As shown in Fig. 1D, ATG7 siRNA pretransfection could effectively inhibit the expression of ATG7. The cells treated by GEM with CQ pretreatment exhibited a greater increase in LC3‐II accumulation, compared to the increase induced by GEM alone (Fig. 1E,F). In contrast, ATG7 siRNA plus GEM resulted in a decrease in GEM‐induced LC3‐II accumulation in PANC‐1 and BxPC‐3 cells (Fig. 1E,F). Meanwhile, the level of p62 was higher in the GEM combined with CQ or ATG7 siRNA group, compared to the GEM group (Fig. 1E,F).

### 3.2 CQ is more effective than ATG7 siRNA in enhancing GEM‐induced cytotoxicity and apoptosis in PC cells

**Figure 2:**
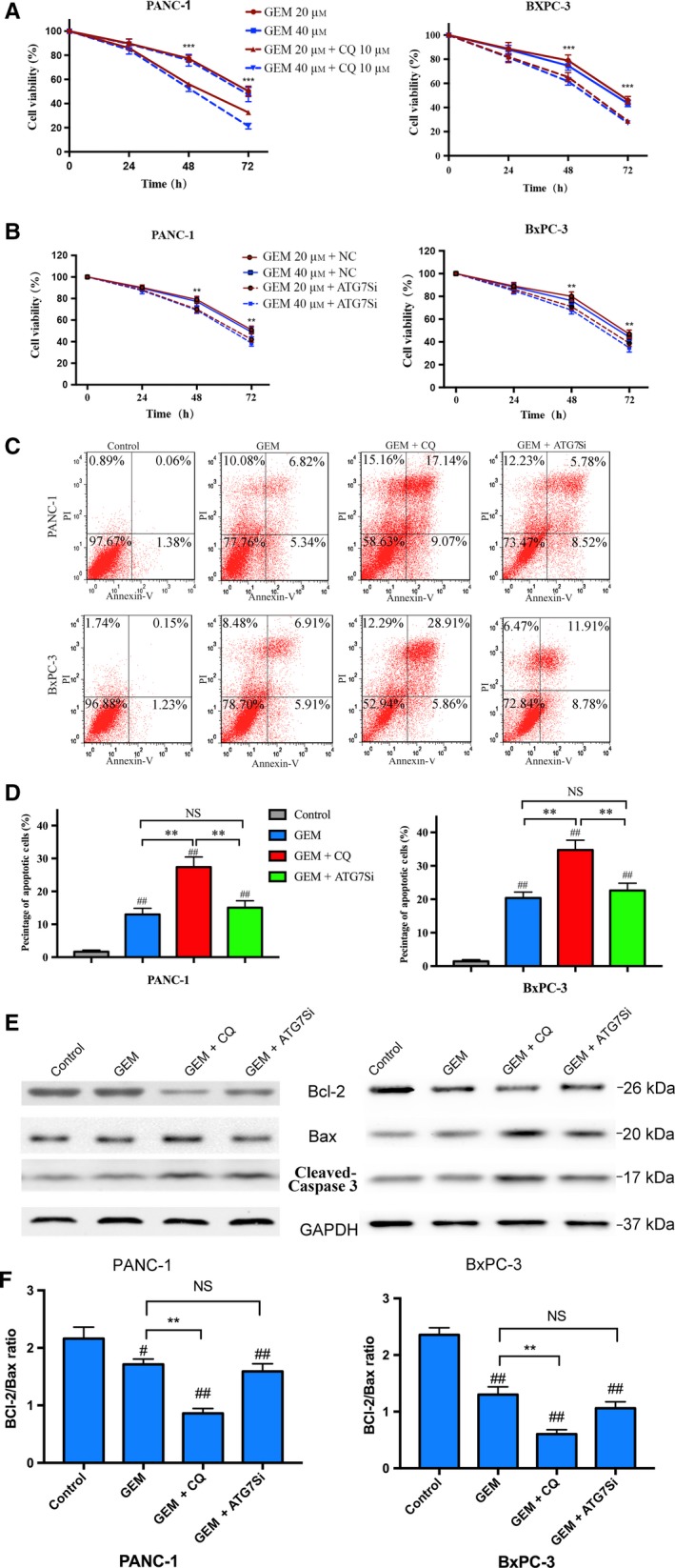
The effects of CQ or ATG7 siRNA on GEM‐induced cytotoxicity and cell apoptosis. (A) In synergistic effect study of CQ combined with GEM, drugs were added at the following concentrations: 0, 20 and 40 μm GEM with/without 10 μm CQ. The cell viability was obtained by assessing the absorbance after incubated for 24, 48 and 72 h. Values are the means of triplicate samples from three independent experiments (±SD). (B) PANC‐1 and BxPC3 cells were transfected with NC siRNA, ATG7 siRNA, respectively, and then treated with GEM. Cell viability was measured using CCK8 assay. **P <* *0.01; ***P <* *0.001,compared with control. (C) Induction of apoptosis in PANC‐1 and BxPC‐3 cells were pretreated with CQ (10 μm) or ATG7 siRNA and then with GEM (20 μm) for another 48 h. The results were representative of three independent experiments. (D) The percentage of apoptotic cells was investigated using Annexin V‐FITC and PI. (AnV+) PI‐ cells were considered early apoptotic, and (AnV+) PI+ cells were considered late apoptotic. The columns represent mean ± SD of three independent experiments. (E) Expression of pro‐apoptotic protein Bax, anti‐apoptotic protein Bcl‐2 and cleaved caspase‐3 were assessed by western blot. The drugs treatments were same as above. The data were representative of three independent experiments. (F) The histograms showed the ratio of Bcl‐2/Bax after different treatments. #*P *<* *0.05; ##*P *<* *0.01, compared with control. NS,* P *>* *0.05; *P <* *0.05; **P <* *0.01.

PANC‐1 and BxPC‐3 cells were pretreated with CQ or pretransfected ATG7 siRNA and then treated with 20 or 40 μmol·L^−1^ of GEM at different time periods (24, 48 and 72 h). The results in Fig. 2A showed that enhanced inhibitory effect on PC cell proliferation by CQ combined with GEM could be observed from the second day in a time‐dependent manner. Cell viability in CQ combined with GEM group was significantly lower than that in GEM group (*P *<* *0.01). When PANC‐1 and BxPC‐3 cells were pretransfected with ATG7 siRNA, we also observed that GEM‐induced cytotoxicity was enhanced from the second day. However, CQ was more effective than ATG7 siRNA in enhancing GEM (20 μmol·L^−1^/48 h)‐induced cytotoxicity in PC cells (PANC‐1: 56.1 ± 2.11% vs 70.0 ± 2.94%, *P *<* *0.01; BxPC‐3: 65.2 ± 3.80% vs 71.0 ± 3.01%, *P *<* *0.05). To further determine whether CQ and ATG7 siRNA could enhance GEM‐induced apoptosis, we used flow cytometry and western blot to detect apoptosis. Using flow cytometry, we observed that GEM could induce apoptosis in both PANC‐1 and BxPC‐3 cells. By CQ pretreatment, the apoptosis induced by GEM could be increased substantially. However, pretransfection with ATG7 siRNA could not significantly enhance the apoptosis induced by GEM in both PANC‐1 and BxPC‐3 cells (Fig. 2C,D). A decreased ratio of Bcl‐2/Bax could be observed by western blot analyses in both PC cell lines after GEM treatment (Fig. 2E,F). Furthermore, GEM administration with CQ pretreatment promoted a decreased ratio of Bcl‐2/Bax and an increase in cleaved caspase‐3 (Fig. 2E,F). GEM combined with ATG7 siRNA pretransfection did not have a similar effect (Fig. 2E,F). Therefore, these results showed that it was CQ, not ATG7 siRNA that could markedly enhance GEM‐induced apoptosis.

### 3.3 GEM with CQ pretreatment or ATG7 siRNA pretransfection can block the cell cycle

**Figure 3:**
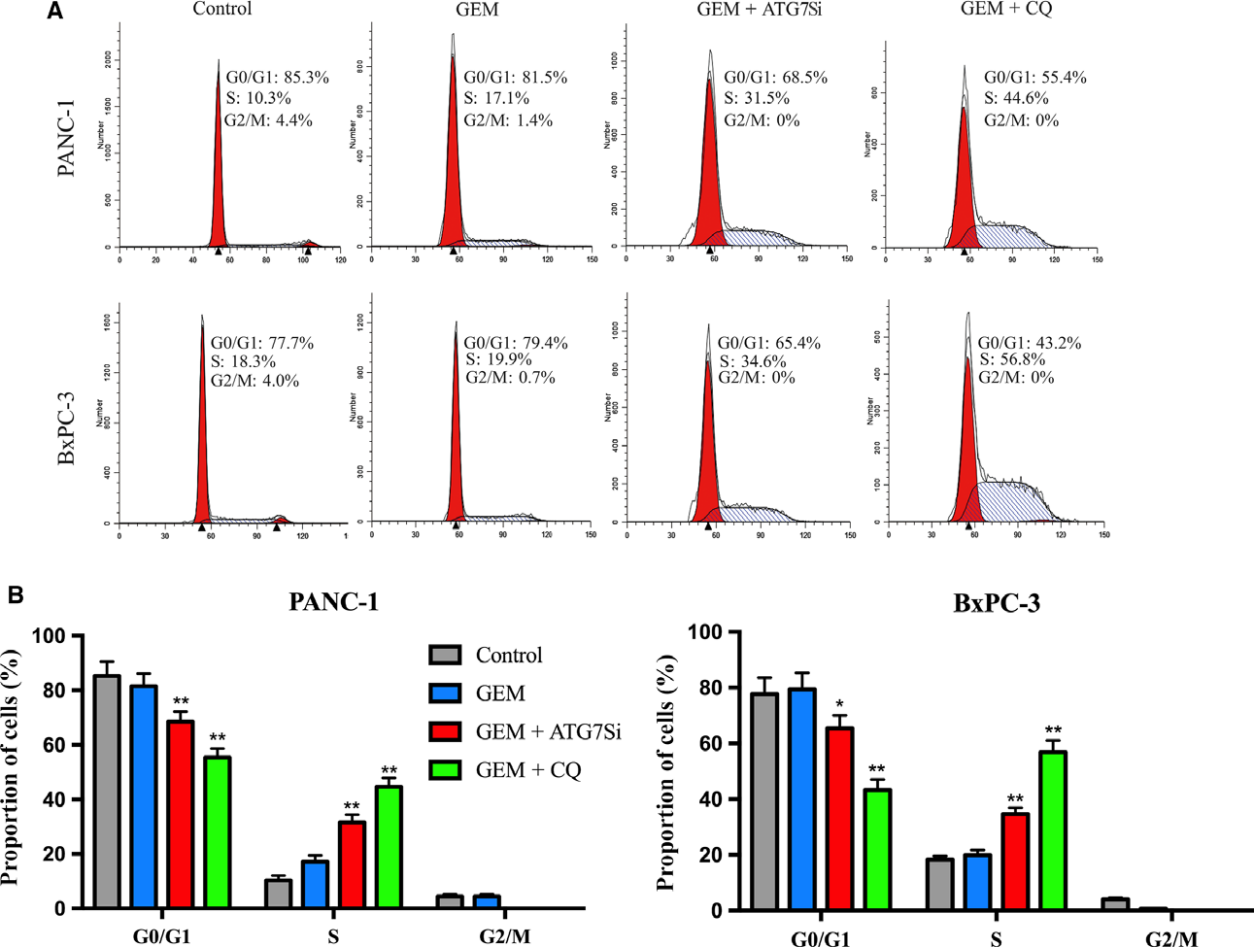
Flow cytometric analysis of cell cycle progression in pancreatic cancer cells treated by GEM with pretreatment of CQ or ATG7 siRNA. (A) Detected cell cycle phase distribution in PANC‐1 and BxPC‐3 cells pretreated with CQ (10 μm) or ATG7 siRNA and then with GEM (20 μm) for another 48 h by flow cytometry. The results were representative of three independent experiments. (B) The percentages of cells in G1, S and G2‐M are shown as histograms. *P <* *0.05; **P <* *0.01, compared with control.

We then analysed cell cycle progression in PANC‐1 and BxPC‐3 cells after GEM treatment combined with CQ or ATG7 siRNA by flow cytometry (Fig. 3A). The percentage of cells in S phase increased significantly, and we could not detect cells in G2/M phase in GEM combined with CQ pretreatment or ATG7 siRNA pretransfection. The percentage of cells in S phase in GEM combined with CQ was significantly greater than GEM combined with ATG7 siRNA (PANC‐1: 44.6 ± 3.3% vs 31.5 ± 2.8%, *P *<* *0.01; BxPC‐3: 56.8 ± 4.3% vs 34.6 ± 2.3%, *P *<* *0.01) (Fig. 3B). In all, these results revealed that CQ pretreatment could effectively postpone the cell cycle of PC cells following GEM application.

### 3.4 ROS production involved in apoptosis induced by GEM combined with CQ in PC

**Figure 4:**
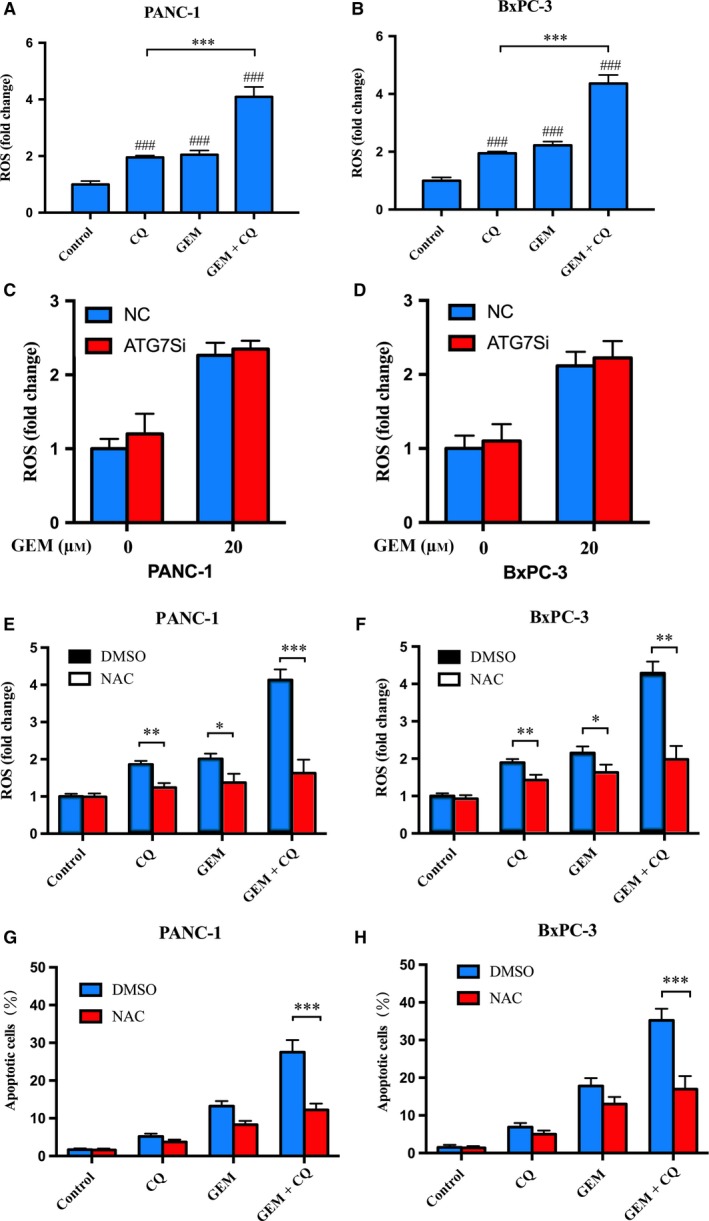
Chloroquine enhanced GEM‐induced apoptosis through ROS production. (A) and (B) PANC‐1 and BxPC‐3 cells were pretreated with CQ (10 μm) for 6 h and then with GEM (20 μm) for another 48 h. The level of ROS production, corresponding to the DCFH‐DA fluorescence intensity, was measured by a fluorometry assay. The levels of ROS were presented as fold change compared to the control group. Values are the means of triplicate samples from three independent experiments. (C) and (D) PANC‐1 and BxPC‐3 cells were transfected with NC siRNA and ATG7 siRNA, respectively, and then were treated with GEM (20 μm) for another 48 h. (E) and (F) PANC‐1 and BxPC‐3 cells were pretreated with NAC (10 μm) for 1 h and then incubated with different treatment. (G) and (H) PANC‐1 and BxPC‐3 cells were pretreated with NAC (10 μm) for 1 h before CQ and GEM treatment. Apoptosis was assessed by an Annexin V‐FITC/PI staining assay. These experiments were repeated in triplicate. ###*P *<* *0.001, compared with control. NS,* P *>* *0.05; *P <* *0.05, **P <* *0.01, ***P <* *0.001.

Chloroquine could remarkably enhance GEM‐induced apoptosis, while ATG7 siRNA failed. The results above showed that CQ‐inhibited autophagy was not essential for the significant induction of apoptosis by GEM combined with CQ. To further recognize the pathway associated with GEM‐induced cell death enhanced via CQ pretreatment, intracellular ROS generation was detected with or without CQ pretreatment. As shown in Fig. 4A,B, the levels of ROS induced by GEM with CQ pretreatment were much higher than those of GEM (PANC‐1: 4.09 ± 0.35 vs 2.05 ± 0.15, *P *<* *0.001; BxPC‐3: 4.36 ± 0.30 vs 2.22 ± 0.13, *P *<* *0.001) (Fig. 4B). In contrast, pretransfection of ATG7 siRNA failed to show the similar effects in both PC cells (Fig. 4C,D, *P* > 0.05).

To further examine whether ROS were involved in apoptosis induced by GEM combined with CQ, we pretreated PANC‐1 and BxPC‐3 cells with the ROS scavenger N‐acetyl‐l‐cysteine (NAC). As shown in Fig. 4E,F, NAC could effectively block the induction of ROS by GEM combined with CQ. As expected, NAC could significantly reduce apoptosis induced by GEM combined with CQ (PANC‐1: 12.24 ± 1.65% vs 27.54 ± 3.19%, *P *<* *0.01; BxPC‐3: 16.98 ± 3.47% vs 35.21 ± 3.09%, *P *<* *0.01) (Fig. 4G,H). The results above suggested that ROS activated by CQ could boost the incidence of GEM‐induced apoptosis.

### 3.5 Cooperation of GEM and CQ enhances lysosomal membrane permeabilization through ROS

**Figure 5:**
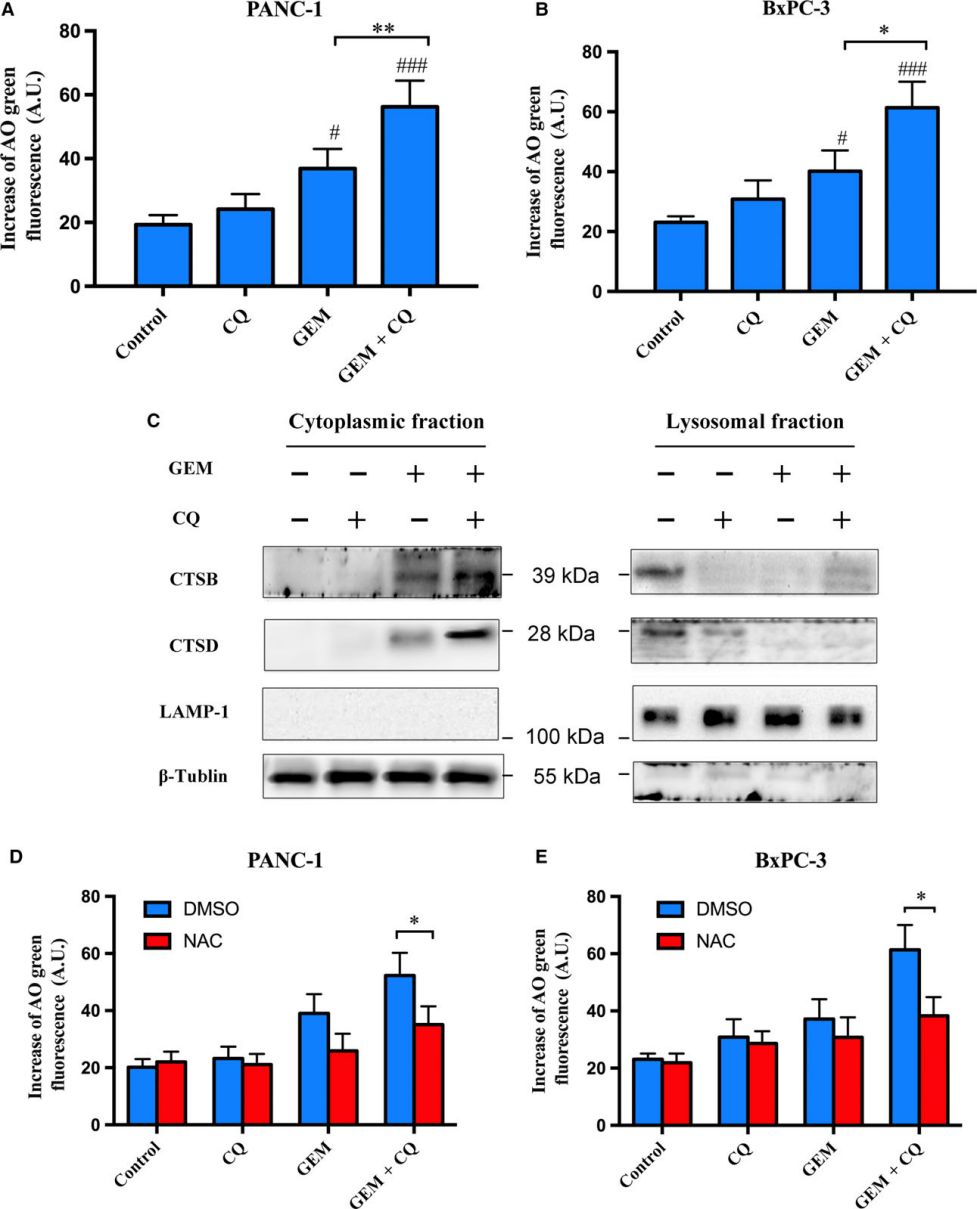
Gemcitabine combined with CQ induced lysosomal membrane permeabilization through ROS production. (A) and (B) The levels of lysosomal membrane permeabilization (LMP) were measured with acridine orange (AO) staining by fluorometry assay. (C) PANC‐1 cells were treated with GEM (20 μm) for 48 h with/without pretreatment of CQ (10 μm) for 6 h. The levels of cathepsin B (CTSB) and cathepsin D (CTSD) from cytoplasmic or lysosomal fractionation were analysed by western blot. The fractionation quality was assessed by the specific subcellular markers: LAMP‐1 for lysosome and β‐tubulin for cytosol. The results were representative of three independent experiments. (D) and (E) PANC‐1 and BxPC‐3 cells were pretreated with NAC (10 μm) for 1 h and then were treated with GEM and CQ either alone or in combination. The levels of LMP were measured with AO staining by fluorometry assay. These experiments were repeated in triplicate. #*P *<* *0.05; ##*P *<* *0.01, compared with control. *P <* *0.05; **P <* *0.01.

The high concentration of ROS may influence lysosomal membrane, and lysosome dysfunction is associated with programmed cell death (Degtyarev *et al*., 2008; Yu *et al*., 2013). We detected lysosomal membrane permeabilization (LMP) after GEM treatment with or without CQ pretreatment. The level of LMP was measured with acridine orange (AO) staining by flow cytometry, and we found that GEM combined with CQ markedly increased LMP more effectively than single drug in both PANC‐1 and BxPC‐3 cells (Fig. 5A,B). The increased LMP may give rise to lysosomal proteases releasing into the cytoplasm, such as cathepsins (Boya and Kroemer, 2008). Therefore, to further confirm a change in LMP induced by GEM with or without CQ, the expression of CTSB and CTSD in the cytoplasm and lysosome was quantitatively detected by western blot. We found that GEM combined with CQ greatly increased the levels of CTSB and CTSD in the cytoplasm. In contrast, the lysosomal CTSB and CTSD levels were significantly reduced by GEM combined with CQ (Fig. 5C). To investigate the effect of ROS on LMP, PANC‐1 and BxPC‐3 cells were pretreated with NAC prior to CQ and GEM treatment. As shown in Fig. 5D,E, NAC significantly reduced LMP in both cells after treatment with both agents. Thus, our results suggested that the enhanced apoptotic effects by GEM and CQ were associated with ROS production.

### 3.6 Cathepsins are involved in apoptosis induced by GEM combined with CQ

**Figure 6:**
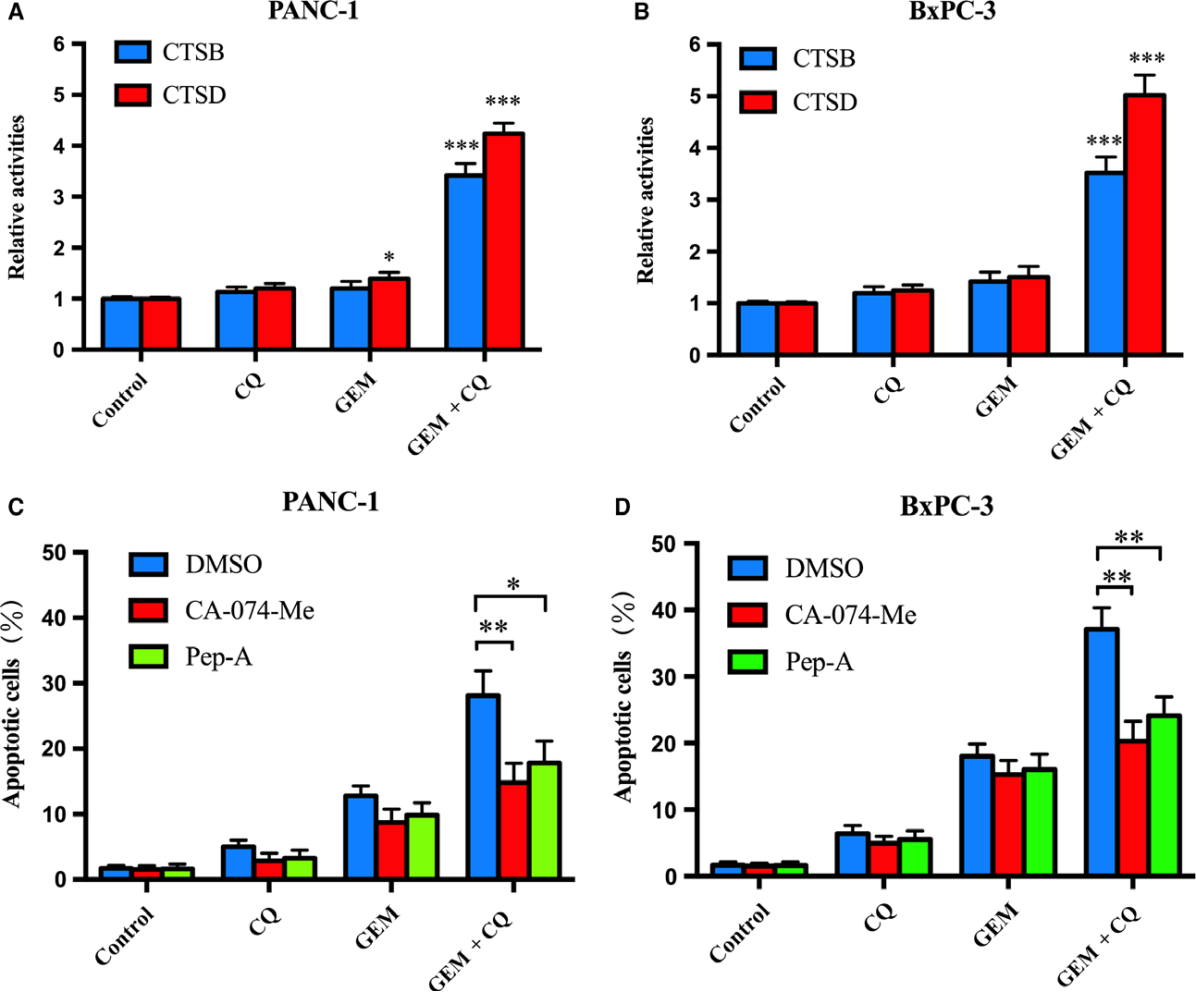
Gemcitabine and CQ in combination treatment induced cell apoptosis involved in cathepsins. (A) and (B) Enzymatic activity of CTSB and CTSD in PANC‐1 and BxPC‐3 cells treated with GEM and CQ either alone or in combination was assessed as described under Section 2. This experiment was repeated in triplicate. (C) and (D) PANC‐1 and BxPC‐3 cells were pretreated with CA‐074‐Me (10 μm) and pepstatin A (Pep‐A) (10 μm) for 1 h, and then cells were treated with GEM and CQ. Apoptosis was assessed by an Annexin V‐FITC/PI staining assay. These experiments were repeated in triplicate. *P <* *0.05; **P < 0.01, ***P < 0.001.

The results shown above demonstrated that CQ in combination with GEM could increase LMP, which resulted in the release of cathepsins into the cytoplasm. As increased lysosomal cathepsin activity in cytoplasm is associated with apoptosis (Bjorkoy *et al*., 2009), the activities of CTSB and CTSD were measured to elucidate the possible mechanism underlying the apoptosis induced by the treatment with GEM and CQ. The levels of active CTSB and CTSD in the cytoplasm were significantly increased in PC cells treated with the two agents in combination (Fig. 6A,B). Next, CA‐074 methyl ester (CA‐074 Me, an inhibitor of CTSB) and pepstatin A (pep‐A, an inhibitor of CTSD) were used to further confirm the interaction between increased cathepsin activities and apoptosis. We found that CA‐074 Me and pep‐A both markedly reduced apoptosis induced by GEM combined with CQ (Fig. 6C,D). The results above showed that increased cathepsin activity in the cytoplasm may cause enhanced apoptosis induced by GEM and CQ in combination.

### 3.7 CQ and GEM synergistically inhibit tumour growth in PANC‐1 tumour xenografts

**Figure 7:**
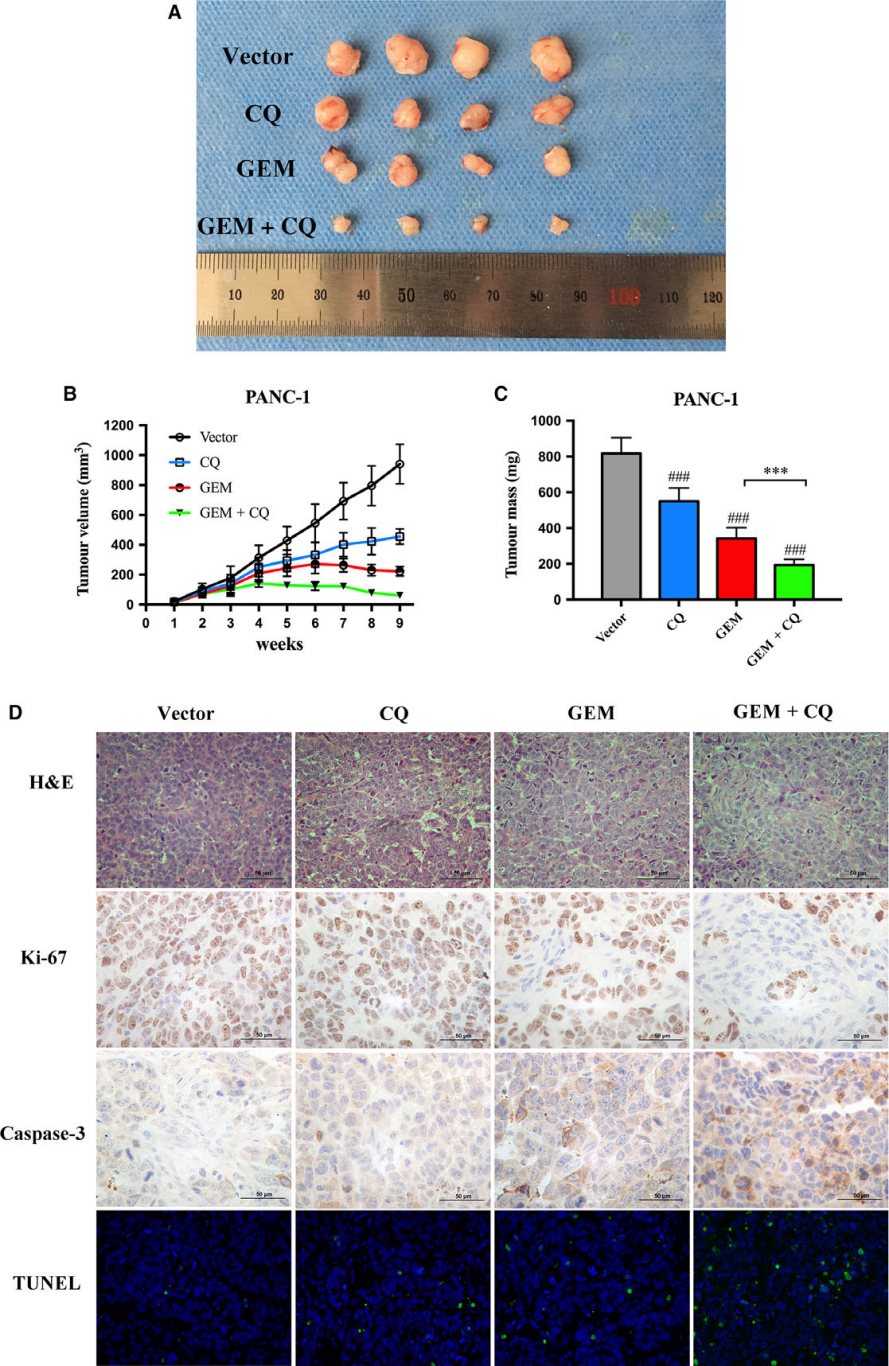
Inhibition of tumour growth *in vivo* by GEM and CQ. (A) Typical images of xenograft tumours. (B) Xenograft tumour volumes were measured twice per week. (C) Average weight of xenograft tumours in nude mice after 4 weeks of growth. (D) Representative images of IHC staining of Ki‐67 and caspase‐3 were performed on serial sections of tumours from PANC‐1/vector, PANC‐1/CQ, PANC‐1/GEM, and PANC‐1/GEM plus CQ group. And cell apoptosis was assessed by TUNEL assay.

Based on the results above, xenograft models were employed to further confirm the enhancing effects of CQ on tumour inhibition. PANC‐1 cells were subcutaneously injected into the right flank of athymic nude mice (Fig. 7A). As expected, the tumours in the CQ‐combined GEM group were significantly smaller than those in the GEM group after 5 weeks of observation. The CQ‐combined GEM group exerted greater antitumour effects in PANC‐1 xenograft tumour models compared with the drugs independently (GEM+CQ vs. GEM: 61.64 ± 8.68 vs. 222.47 ± 31.33 mm^3^, *P *< 0.01; GEM+CQ vs. CQ: 61.64 ± 8.68 vs. 455.88 ± 51.85 mm^3^, *P *<* *0.01) (Fig. 7B). Consistent with the results of tumour volume, tumour weight could be suppressed by GEM monotherapy or CQ+GEM combination therapy. GEM combined with CQ pretreatment showed a more effective inhibition in pancreatic inoculated mice (GEM + CQ vs. GEM: 194.13 ± 31.10 mg vs. 342.25 ± 60.58 mg, *P *<* *0.001) (Fig. 7C). Moreover, Ki‐67 expression was markedly reduced in xenograft tumours in the CQ‐combined GEM group compared with other groups (Fig. 7D). The levels of caspase‐3 were significantly increased in the CQ‐combined GEM group, and the boosted apoptosis activation was also confirmed by TUNEL assay. The results above showed that GEM combined with CQ inhibited PC cell proliferation and induced apoptosis *in vivo*. Altogether, the data from *in vitro* and *in vivo* assays confirmed that CQ pretreatment could significantly increase chemosensitivity to GEM for PC cells.

## 4 Discussion

Pancreatic cancer is one of the most lethal human malignancies in the world, and its poor prognosis makes the requirement for more effective treatment. Many patients with PC are in an advanced stage when they are diagnosed. Even if the tumour is radically resected, recurrence and metastases make prognosis still poor. GEM, the nucleoside analogue, is currently the leading therapeutic for PC treatment (Hidalgo, 2010). However, due to the growing resistance to GEM, recognizing the mechanisms that underlie GEM resistance and discovering agents that increase tumour sensitivity to GEM could be an important key to improving the prognosis of PC (Oettle *et al*., 2007).

Autophagy is a dynamic process involving the bulk degradation of cytoplasmic organelles and proteins. Based on the function of cellular recycling, autophagy plays a key role in the quality control of cellular components as well as supplying nutrients and materials for newly constructed structures in cells under metabolic stress (Tsuchihara *et al*., 2009). Recent studies suggest that autophagy may be important not only in the regulation of cancer development and progression but also in determining the response of tumour cells to anticancer therapy (Du *et al*., 2012; Pardo *et al*., 2010). In this study, we found that both the number of autophagic vacuoles increased after GEM treatment and GEM‐induced autophagy can be effectively abolished by CQ or ATG7 siRNA. The inhibition of autophagy can sensitize PC cells to the antiproliferative effect caused by GEM, which suggested that GEM‐induced autophagy may play a protective role in chemotherapy resistance. On the other hand, although both ATG7 siRNA and CQ could inhibit autophagy, CQ had stronger synergistic effects than ATG7 siRNA in GEM‐induced cytotoxicity. The differences between CQ and ATG7 siRNA suggested that other mechanisms, in addition to autophagy, could be involved in GEM chemotherapy resensitizing.

Chloroquine has been proven to be an effective agent for breast cancer and osteosarcoma, and there is empirical evidence that CQ may induce cell death and an increase in caspase‐3 activation in glioma cell lines (Cufi *et al*., 2013; Geng *et al*., 2010; Shen *et al*., 2013). CQ is also a sensitizer for CoCl_2_‐induced growth arrest and differentiation of leukaemic cells (Yan *et al*., 2013). Recently, some researches have applied GEM combined with CQ to metastatic or unresectable PC (Boone *et al*., 2015; Samaras *et al*., 2017). The primary results showed promising safety and biologic response. However, the efficacy and underlying mechanisms required further assessment. In our study, evidenced by flow cytometry and western blot, we confirmed that CQ pretreatment, instead of ATG7 siRNA, could significantly enhance GEM‐induced apoptosis in addition to autophagy prohibition. Therefore, our results suggested that not only autophagy inhibition but also apoptosis activation markedly contributed to GEM resensitizing by CQ in PC cells. As a vital mediator of apoptosis, ROS have been identified as signalling molecules in various pathways regulating both cell survival and cell death. Numerous stimuli, including hypoxia, nutrient starvation and chemostress, can induce autophagy and ROS (Scherz‐Shouval and Elazar, 2007). In our study, CQ pretreatment could markedly increase ROS induced by GEM, while ATG7 siRNA failed to show the similar effect. Meanwhile, when ROS was inhibited by NAC, apoptosis triggered by GEM with CQ pretreatment could be abolished. Thus, our study illustrated that CQ may increase ROS to promote GEM‐induced apoptosis.

Excessive ROS has been demonstrated to disrupt the lysosomal membrane and cause LMP, which may result in apoptosis (Fukuda, 1991; Redza‐Dutordoir and Averill‐Bates, 2016). Evidenced by living cell AO staining and increased levels of CTSB/CTSD in cytosolic extracts, we confirmed that GEM combined with CQ could markedly induce LMP in PC cells. Those effects could be restored by NAC, suggesting the key role of ROS in LMP induction by GEM combined with CQ. Increased lysosomal cathepsin activity in cytoplasm was associated with apoptosis (Kundra and Kornfeld, 1999); therefore, increased CTSB/CTSD levels indicated the possible connection between GEM combined with CQ pretreatment and apoptosis induction after LMP. The quantitative change in Bax in our study indicated that the released CTSB/CTSD may induce Bax and its translocation to the mitochondria, which may subsequently promote apoptosis (Demers‐Lamarche *et al*., 2016). Interestingly, we found that CQ could induce similar levels of ROS as GEM (Fig. 4C,D). However, CQ could not remarkably increase LMP, while GEM could (Fig. 5A,B). We speculated the possible reason was that PC cells were treated with GEM for 48 h, while treated with CQ only for 6 h. These results indicated that ROS may require some time to cause LMP. Taken together, our findings demonstrated that GEM combined with CQ induced the lysosomal apoptotic pathway through ROS.

As an established anticancer drug, GEM is an effective systemic treatment for PC unfortunately, chemo‐escape and resistance to GEM make it impossible to eliminate all PC cells. Combination with CQ GEM showed the greatest inhibition of tumour xenografts than all other groups. Based on our study, CQ could be used as GEM chemotherapy sensitizer; however, CQ itself was not a chemotherapeutic agent. Halting CQ treatment may decrease GEM chemotherapy effects, and the capability of tumour regrowing largely depends on GEM instead of CQ. Therefore, more researches, such as combination interval and therapeutic window, are required to build up more promising synergistic effects.

In summary, our results showed that CQ can enhance GEM‐induced apoptosis in PC cells *in vitro* and *in vivo*. The increase in ROS induced by GEM combined with CQ may be an upstream event that triggers lysosomal membrane permeabilization. Then, cathepsins are released into the cytoplasm from lysosomes, inducing apoptosis consequently. Our study indicated a role of CQ as a potential adjuvant with GEM in the treatment of PC.

## Author contributions

JZ and QW contributed to designing the research, assembly data and data interpretation. JZ, FZ, CX and KJ performed the major portion of the experiments. FH, CL, LJ and SX performed parts of the research. SB and PC tested statistics and coordinated the figures. JZ and YS wrote the manuscript. QW and SB revised the manuscript.
